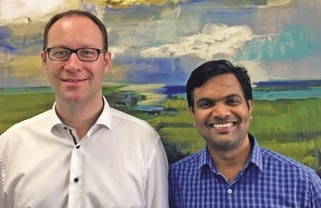# Uncovering the Neglected Similarities of Arynes and Donor–Acceptor Cyclopropanes

**DOI:** 10.1002/anie.201909213

**Published:** 2019-12-12

**Authors:** Daniel B. Werz, Akkattu T. Biju

**Affiliations:** ^1^ Technische Universität Braunschweig Institut für Organische Chemie Hagenring 30 38106 Braunschweig Germany; ^2^ Department of Organic Chemistry Indian Institute of Science Bangalore 560012 India

**Keywords:** annulations, arynes, cyclopropanes, donor–acceptor systems, insertions

## Abstract

Arynes and donor–acceptor (D–A) cyclopropanes are two classes of strained systems having the potential for numerous applications in organic synthesis. The last two decades have witnessed a renaissance of interest in the chemistry of these species primarily because of the mild and robust methods for their generation or activation. Commonly, arynes as easily polarizable systems result in 1,2‐disubstitution, whereas D‐A cyclopropanes as polarized systems lead to 1,3‐bisfunctionalization thereby showing striking similarities. Transformations with 1,2‐ and 1,3‐dipoles afford cyclic structures. With arynes, emerging four‐membered rings as intermediates might react further, whereas the analogous five‐membered rings obtained from D–A cyclopropanes are most often the final products. However, there are a few cases where these intermediates behave surprisingly differently. This Minireview highlights the parallels in reactivity between arynes and D–A cyclopropanes thereby shedding light on the neglected similarities of these two reactive species.

## Graphical abstract

**Two sides of the same coin?** Arynes and donor–acceptor cyclopropanes show striking similarities in their reactivity. This Minireview highlights analogous insertion and annulation reactions of these two strained species.

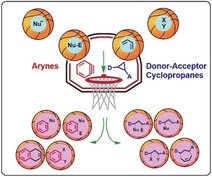

## 1 Introduction

**Figure 1:**
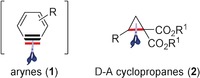
Arynes and D–A cyclopropanes with their reactive bonds (depicted in red).

Construction of carbon–carbon and carbon–heteroatom bonds proceeding via the in situ generation of reactive intermediates has received much attention in organic synthesis. Among the various reactive intermediates, arynes generated from the respective precursors and carbon‐based 1,3‐dipoles formally derived from donor–acceptor (D–A) cyclopropanes have become versatile two‐ and three‐carbon building blocks showing numerous applications. Arynes **1** are highly reactive intermediates, which are widely utilized for the construction of 1,2‐disubstituted benzene derivatives (Figure 1).^1^ The high electrophilicity of arynes has been exploited for the synthesis of monofunctionalized, vicinally di‐ and even trifunctionalized arenes.^2^ The mild generation of this useful intermediate using the fluoride‐induced desilylation method developed by Kobayashi and co‐workers has contributed immensely to its development as a two‐carbon synthon.^3^ In the case of D–A cyclopropanes **2** (this term was originally coined by Reissig),^4^ the vicinal arrangement of the donor and acceptor groups as well as the extraordinary reactivity of the cyclopropane moiety make them a versatile class of three‐atom building blocks.^5^, ^6^, ^7^ There are several cases where arynes and D–A cyclopropanes display similar reactivity towards nucleophiles, dipoles, and diene‐type compounds. Clearly, their reactivity is analogous to that of α,β‐unsaturated carbonyl compounds. The purpose of this Minireview is to highlight the similarities in reactivity of these highly versatile systems by comparing conceptually analogous transformations.

From a historic perspective, arynes were postulated as an intermediate in 1902 by Stoermer and Kahlert.^8^ Moreover, Wittig proposed the benzyne intermediate in 1942 in the reaction of fluorobenzene and phenyl lithium.^9^ Later Roberts et al. used arynes to explain the reactivity of ^14^C‐labeled chlorobenzene in the synthesis of aniline (Table 1, entry 1).^10^ The seminal contributions in the area of D–A cyclopropanes were provided by Wenkert,^11^ Danishefsky^12^ and Reissig et al.^13^ in the late 1970s. These scientists were responsible for developing many of the fundamental reactions of those species. Arynes are two‐carbon synthons employed in various 1,2‐bisfunctionalization reactions, whereas D–A cyclopropanes are three‐carbon entities, which could be engaged in several 1,3‐bisfunctionalizations (Table 1, entry 2). Arynes have a low‐lying LUMO due to the strained nature of the formal C−C triple bond embedded in the six‐membered ring. The respective strain energy was calculated to be 63 kcal mol^−1^, making these species highly reactive (Table 1, entry 3).^14^ Moreover, the bond length of the formal C−C triple bond of 1.24 Å in arynes is an indication of partial double‐bond character.^15^ In the case of D–A cyclopropanes, the strain energy of 27.5 kcal mol^−1^ associated with the three‐membered ring is the driving force for the ring‐opening under mild conditions. Whereas arynes are highly reactive and have to be generated in situ under the reaction conditions (Table 1, entry 4), D–A cyclopropanes are bench‐stable and commonly require activation by Lewis acids for enhanced reactivity.

**Table 1:** Comparison of arynes and D–A cyclopropanes.

Entry	Arynes	D–A Cyclopropanes
1	early observations: Stoermer (1902), Wittig (1942)	early work: Wenkert, Danishefsky and Reissig in the late 1970s
2	two‐carbon synthons useful for 1,2‐bisfunctionalization	three‐carbon units useful for 1,3‐bisfunctionalization
3	strained C−C triple bond: low‐lying LUMO with a strain energy of 63 kcal mol^−1^. Release of strain is key for reactivity.	strained C−C single bond: highly reactive with a strain energy of 27.5 kcal mol^−1^. Release of strain is key for reactivity.
4	generated in situ: activation is commonly not required	stable: activation is commonly required
5	polarizable system	polarized system; polarization is increased by Lewis acids
6	reactions suffer from poor regioselectivity	reactions are commonly highly regioselective

Arynes are non‐polar species, but the formal triple bond is easily polarizable in the presence of a nucleophile(Table 1, entry 5). The push–pull effect of the adjacent electron‐donating and electron‐accepting units in D–A cyclopropanes induces a high degree of polarization of the C−C single bond, which can be even further enhanced in the presence of Lewis acids. Transformations of unsymmetrically substituted arynes give rise to the question to which degree regioselectivity is obtained (Table 1, entry 6). Although there have been several in‐depth investigations over the last decade by both, experimental and computational means, common product ratios are often in the range of 1:1 to 5:1.^16^ This situation is completely different in the case of donor–acceptor cyclopropanes. Regioselectivity is not an issue at all. The strongly polarized system allows a completely regioselective attack of the nucleophile. However, in the case of the ring‐opening of the D–A cyclopropanes, the question is whether the original stereochemistry is retained, inverted, or whether there is a complete loss of stereochemical information. Whereas the 1,2‐disubstituted arenes being generated from arynes are perfectly stable compounds, respective 1,3‐bisfunctionalized products being obtained from aryl‐substituted D–A cyclopropanes suffer in some cases from the possibility to undergo elimination reactions affording styrene derivatives. These emerging olefinic compounds might further react with D–A cyclopropanes and give rise to a larger variety of products than originally anticipated.^17^

Arynes and D–A cyclopropanes resemble each other in several annulations and 1,*n*‐bisfunctionalization reactions (*n*=2, 3). Arynes react via the cleavage of the weak in‐plane π‐bond of the formal C−C triple bond, whereas D–A cyclopropanes react via the cleavage of the labile C−C σ‐bond (which has considerable π‐character) under the influence of Lewis acids. In both cases, the driving force is the release of strain energy.

In this Minireview, it is not our aim to provide a comprehensive overview of the plethora of transformations that have been carried out using arynes and D–A cyclopropanes. We will limit ourselves to the most striking examples that demonstrate the similarities in the reactivity of these two species, which have recently enjoyed such a renaissance in the synthetic organic community.

## 2 Insertion Reactions

**Scheme 1:**
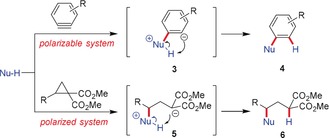
Insertion of arynes (top) and D–A cyclopropanes (bottom) into a Nu−H bond. Nu=nucleophile.

Both, arynes and D–A cyclopropanes, are unsaturated species. While the formal triple‐bond system of the aryne is transformed into a C−C double bond by a 1,2‐addition leading to a 1,2‐disubstituted arene, the weakest C−C single bond of the cyclopropane might be cleaved leading to a ring‐opening and thus to 1,3‐bisfunctionalized products. The simplest reagents that are able to undergo these transformations are nucleophiles Nu‐H such as amines, alcohols, and thiols. The easily polarizable formal triple bond of the aryne is attacked by nucleophilic species, generating intermediate **3**. The developing negative charge is neutralized by a proton transfer from the former nucleophilic carbon or heteroatom to afford the inserted product **4** (Scheme 1). This scenario is paralleled in the case of D–A cyclopropanes. The positively polarized carbon atom next to the donor unit is attacked by a nucleophile, generating intermediate **5**, while the emerging negative charge at the substituted malonate is neutralized by a proton transfer to furnish **6**.

### 2.1 Insertion into O−H, S−H, N−H, and C−H Bonds

**Scheme 2:**
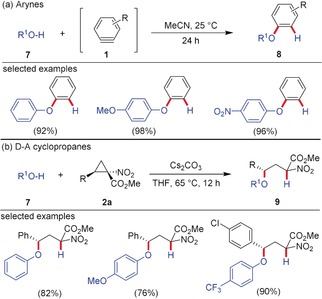
Insertion of a) arynes and b) D–A cyclopropanes into phenolic O−H bonds.

Both arynes and D–A cyclopropanes insert into O−H bonds. In aryne chemistry, this method has become a powerful tool to synthesize aryl and diarylethers **8** by the insertion of alcohols/phenols **7**, and has been extended to the corresponding sulfur analogs (Scheme 2).^18^ This chemistry has been paralleled when alcohols or thiols were used for the ring‐opening of D–A cyclopropanes. (Thio)phenols were successfully employed and yielded products **9** with a novel C−O (C−S) bond under inversion of configuration.^19^ Under different conditions, even aliphatic alcohols were successfully employed for these reactions. Interestingly, the change from phenol to naphthol or naphthoquinone dianions did not lead to C−O bond formation in the reaction with D–A cyclopropanes, but to C−C bond formation.^20^ Such C−C coupling with naphthol derivatives has never been observed in the case of arynes. This result showcases that subtle differences in the nucleophilicity might lead to a completely different outcome. Arynes were also inserted into the O−H bond of carboxylic acids.^18^ The nucleophilic carboxylate readily attacks the low‐lying LUMO of the aryne, and aryl esters are obtained. It seems that the delocalized negative charge of a carboxylate does not allow a facile attack of a D–A cyclopropane; only one example is known where a D–A cyclopropane is formally inserted into the O−H bond of a carboxylic acid. In this case, a large excess of TFA was used to yield that product with several other by‐products in low yield.^17^

**Scheme 3:**
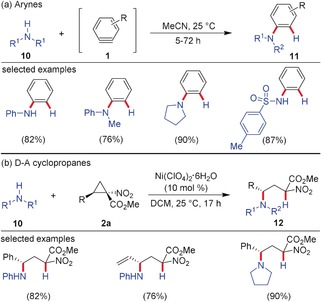
Insertion of a) arynes and b) D–A cyclopropanes into different types of N−H bonds. DCM=dicholoromethane.

Similarities are also observed in N−H insertion reactions. The arylation of primary/secondary amines **10** and sulfonamides by arynes has become a straightforward method to synthesize the corresponding arylated counterparts **11** in good to excellent yields (Scheme 3).18b D–A cyclopropanes **2 a** with a NO_2_ group and a carboxylic ester as accepting moieties were transformed with aniline derivatives and cyclic secondary alkyl amines such as pyrrolidine and piperidine into the respective 1,3‐ring‐opened products **12** in good yields.^21^ Other alkylated amines proved to be too nucleophilic and shut down the reaction by complexation of the Lewis acid, which is needed to activate the cyclopropanes. Stereochemical information at the reactive center was conserved in reactions using chiral cyclopropanes with inversion in stereochemistry.

**Scheme 4:**
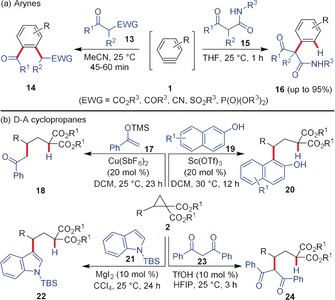
Insertion of a) arynes and b) D–A cyclopropanes into different types of C−H bonds. EWG=electron‐withdrawing group, HFIP=hexafluoro‐2‐propanol, TfOH=trifluoromethanesulfonic acid, TBS=*tert*‐butyldimethylsilyl, TMS=trimethylsilyl.

With respect to the insertion of arynes and D–A cyclopropanes into C−H bonds there are similarities, but also striking differences. The Stoltz and Yoshida groups independently reported that arynes react with β‐ketoesters or 1,3‐dicarbonyls **13** to give the acyl‐alkylation product **14** resulting from a C−C bond cleavage of the activated dicarbonyl compound followed by insertion (Scheme 4).^22^ Mechanistically, one assumes an initial nucleophilic attack of the enolate on the triple bond of the aryne, ring‐closure to a four‐membered ring, and fragmentation to the 1,2‐disubstituted benzene derivative. Such a cleavage of an enol C−C bond has never been observed so far in the reaction with D–A cyclopropanes. However, this common pathway can be interrupted when the substrate bears a proton source, for example, when β‐keto amides **15** are utilized. Then, the α‐arylated products **16** are formed.^23^

Substrates with strongly nucleophilic carbon atoms have also been employed to open D–A cyclopropanes. Tang and co‐workers reported the Cu‐catalyzed addition of silyl enolethers **17** to D–A cyclopropanes in the absence of ligands to furnish the alkylated product **18** in good yields.^24^ Moreover, the addition of 2‐naphthols **19** to D–A cyclopropanes resulting in the Friedel–Crafts product **20** was demonstrated by Biju and co‐workers.20a In addition, the reaction of indoles **21** with D–A cyclopropanes in the presence of MgI_2_ catalyst and a pybox ligand resulted in the dynamic kinetic Friedel–Crafts alkylation reaction to afford **22** in high yield and *ee* values.^25^ Furthermore, the TfOH‐catalyzed reaction of dibenzoyl methane **23** with D–A cyclopropanes resulting in the formation of α‐alkylated product **24** was disclosed by Moran and co‐workers.^26^

### 2.2 1,2‐ and 1,3‐Bisfunctionalizations

**Scheme 5:**
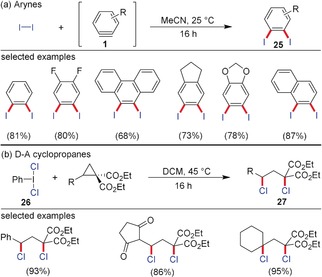
a) 1,2‐Diiodination of arynes using molecular iodine and b) 1,3‐bischlorination of D–A cyclopropanes using PhICl_2_.

As shown in Section 2.1, formal insertions of arynes into polarized X−H bonds proceed very easily. The same holds true for the nucleophilic ring‐opening of D–A cyclopropanes. The situation becomes more complicated when two substituents are attached to an aryne (and none of them must be hydrogen) and when ring‐opening reactions of D–A cyclopropanes are conducted leading to 1,3‐bisfunctionalized motifs. One needs to differentiate between the same type of substituents attached in 1,2‐ and 1,3‐positions, or two different types. For the same type of substituents there are examples of the attachment of halides to arynes and D–A cyclopropanes. Molecular iodine as an easily polarizable dihalogen is able to attack the strained triple bond. Although the question of whether a real nucleophile (iodide) or molecular iodine approaches the aryne has not been elucidated, this procedure has become a very useful method to generate 1,2‐diiodobenzene derivatives **25** (Scheme 5).^27^ With D–A cyclopropanes the analogous 1,3‐bisiodination has not been realized; however, a similar 1,3‐bischlorination method was developed by Werz and co‐workers using Willgerodt′s reagent PhICl_2_ (**26**) as the chlorine source to afford **27**.^28^ Mechanistic experiments suggested that the three‐membered ring is opened in a radical‐like fashion with the first Cl radical on the carbon atom substituted by the donor. Because of the radical mechanism no Lewis acid catalyst to coordinate the electron‐withdrawing groups is needed; therefore, even electron‐withdrawing groups such as ketones, nitriles, and nitro groups are tolerated. Besides aryl systems, oxygen, nitrogen, and even aliphatic groups as donors are also possible.

**Scheme 6:**
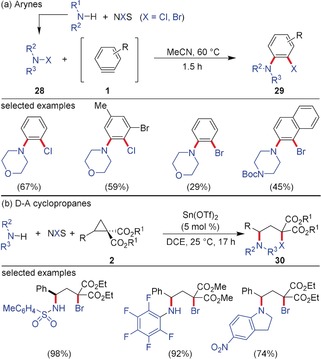
a) 1,2‐Aminochlorination and ‐bromination of arynes and b) 1,3‐aminobromination of D–A cyclopropanes using a three‐component approach. Boc=*tert*‐butoxycarbonyl, DCE=1,2‐dichloroethane, NXS=*N*‐chloro‐ or *N*‐bromosuccinimide (NBS).

More challenging are 1,2‐ or 1,3‐bisfunctionalizations of arynes or D–A cyclopropanes, respectively, with two different substituents. These reactions can be either conducted with a highly polarized system X–Y or in a three‐component approach. The latter approach is often hampered by a direct reaction of the nucleophile with the electrophile without involving the aryne or the D–A cyclopropane in the desired transformation. 1‐Chloro‐ and 1‐bromo‐2‐amino‐arenes were accessed by aryne chemistry using a three‐component approach (Scheme 6).^29^ The assumption is that *N*‐chloro‐ or *N*‐bromosuccinimide react first with the respective secondary amines. The generated *N*‐haloamines **28** show sufficient nucleophilicity to attack the strained triple bond, while the emerging vinyl anion is captured by the positively polarized halogen to afford the amino‐arylated product **29**. Similar products were obtained via the attack of imines on the aryne and the chlorination at the 2‐position by carbon tetrachloride, while the emerging CCl_3_ anion attacked the sp^2^‐hybridized carbon of the heterocarbonyl.^30^ A related reaction was realized by the Studer group for D–A cyclopropanes during the synthesis of 1,3‐aminohalogenated product **30**.^31^ As nucleophilic component, sulfonamides or primary amines with strongly electron‐withdrawing groups had to be employed. These species are able to open the three‐membered ring after activation with Sn(OTf)_2_. As electrophilic halogen component, NBS comes into play capturing the emerging negatively charged malonate. An analogous reaction was recently realized with *N*‐(phenylthio)‐ and *N*‐(phenylseleno)succinimides as electrophilic component leading to a ring‐opening 1,3‐aminochalcogenation.^32^ With sulfonamides, the corresponding ring‐opened product was also detected, but in very low yield. In the field of arynes, the S−N insertion was developed by Biju and co‐workers using sulfenamides.^33^ The weak N−S σ‐bond is easily broken and 1,2‐sulfanyl anilines are obtained.

## 3 Annulation Reactions

Arynes and D–A cyclopropanes resemble each other in a variety of annulation reactions. Both entities react with dienes, 1,3‐dipoles, and electrophiles such as aldehydes and imines to form annulated products. Arynes react as a 2π‐component leading to various (2+2)‐, (3+2)‐, (4+2)‐annulations, whereas the labile C−C bond in cyclopropanes can react by (3+2)‐, (3+3)‐, and (3+4)‐pathways. A series of benzo‐fused heterocycles are formed with arynes, and several five‐ to seven‐membered carbo‐ and heterocycles are accessible using D–A cyclopropanes.

### 3.1 Reaction with Dienes and Heterodienes

**Scheme 7:**
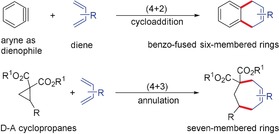
Reaction of arynes and D–A cyclopropanes with dienes.

The low‐lying LUMO of arynes turns them into good dienophiles, which can be easily intercepted by dienes in Diels–Alder reactions. Aryne Diels–Alder reactions are often used as a method for the detection of aryne intermediates as well for the synthesis of functionalized arenes (Scheme 7). Like arynes, the cyclopropane ring activated by Lewis acids can be opened using dienes in a (4+3)‐annulation reaction leading to seven‐membered rings. This [4π+2σ]‐annulation might be regarded as a formal analog of the Diels–Alder reaction.

**Scheme 8:**
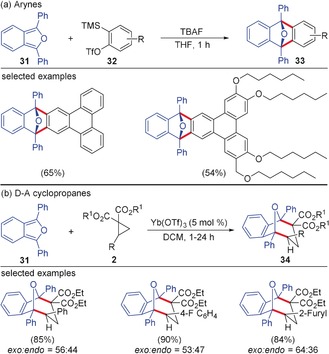
Reaction of a) arynes and b) D–A cyclopropanes with 1,3‐diphenylisobenzofuran. TBAF=tetrabutylammonium fluoride.

Arynes undergo Diels–Alder reactions with a series of acyclic and cyclic dienes.1g Aryne Diels–Alder reactions pave the way for the preparation of various polycyclic aromatic hydrocarbons for applications in materials chemistry.^34^ For instance, in 2006, Pérez, Peña and co‐workers reported the Diels–Alder reaction of 1,3‐diphenylisobenzofuran **31** with arynes generated from the triflate precursor **32** resulting in the formation of polycyclic compounds of type **33** in good yields (Scheme 8).^35^ Analogous to the reactivity with arynes, D–A cyclopropanes underwent smooth (4+3)‐annulation with 1,3‐diphenylisobenzofurans for the synthesis of cycloadduct **34** in good yields. The use of Yb(OTf)_3_ as Lewis acid in CH_2_Cl_2_ under refluxing conditions was required for better conversion.^36^ Similar annulation reactions of arynes and D–A cyclopropanes with anthracene as the diene component have also been realized.^37^ In this context it is noteworthy that although aryne Diels–Alder reactions with pentafulvenes leading to benzonorbornadiene derivatives are known,^38^ related annulation processes using D–A cyclopropanes have not been reported.

**Scheme 9:**
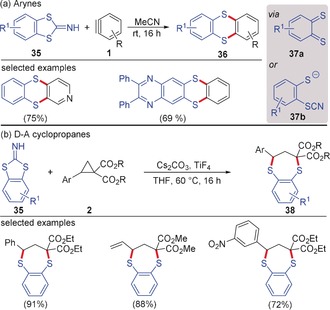
a) Reaction of arynes and b) D–A cyclopropanes with benzodithioloimine as *ortho*‐bisthiobenzoquinone surrogate.

Similar trends in reactivity were observed with heterodienes. Werz and co‐workers disclosed the formal Diels–Alder reaction of *ortho*‐bisthiobenzoquinones **37 a** with arynes resulting in the formation of thianthrene derivatives **36** (Scheme 9).^39^ Although a free heterodiene **37 a** was not considered as an intermediate, the transformation is most easily understood in terms of a (4+2)‐annulation process. The starting material for the sulfur component was benzodithioloimine **35** in which the sulfur shows amphiphilic reactivity. Under the influence of base, the five‐membered ring opens leading to species **37 b** with nucleophilic (thiolate) and electrophilic sulfur (thiocyanate). The (4+2)‐adduct was formed in good yields under transition‐metal‐free conditions.^40^ The Werz group further extended their study to D–A cyclopropanes to demonstrate the similarity in reactivity of the two reactive species, where a smooth (4+3)‐annulation was observed leading to the synthesis of dithiepine derivatives **38**.^41^ A series of aromatic donors and even the amino‐substituted D–A cyclopropanes developed by Waser et al.25b were used in this annulation. Employing enantiomerically pure cyclopropane resulted in the stereospecific (4+3)‐annulation.

### 3.2 Reaction with 1,3‐Dipoles

**Scheme 10:**
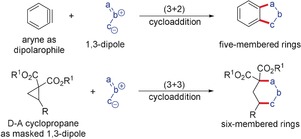
1,3‐Dipolar cycloaddition reactions of arynes and D–A cyclopropanes.

Because of the high electrophilicity of arynes, they are excellent dipolarophiles, and are able to add to various 1,3‐dipoles resulting in the construction of a series of benzo‐fused five‐membered heterocycles (Scheme 10). Most of these reactions work under mild conditions in the absence of any transition‐metal catalyst. Analogous to arynes, D–A cyclopropanes activated by Lewis acid are able to add to 1,3‐dipoles leading to (3+3)‐dipolar cycloaddition reactions resulting in the formation of six‐membered heterocycles.

**Scheme 11:**
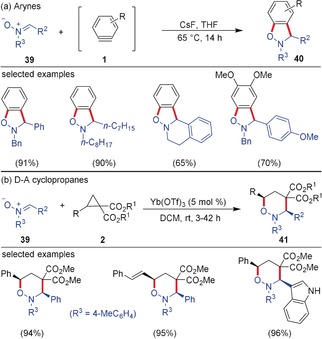
a) (3+2)‐Cycloaddition of arynes and b) (3+3)‐cycloaddition of D–A cyclopropanes with nitrones. Bn=benzyl.

Nitrones are commonly used 1,3‐dipoles employed in (3+2)‐cycloaddition reactions for the synthesis of isoxazoles. As early as 2008, Danishefsky and co‐workers utilized α,β‐unsaturated nitrones as 1,3‐dipoles in (3+2)‐cycloaddition reactions with arynes, and they used this method for the synthesis of the cortistatin core.^42^ Larock and co‐workers demonstrated a facile synthesis of benzisoxazolines **40** by the (3+2)‐cycloaddition of nitrones **39** with arynes. The mild method tolerates a variety of functional groups thereby providing access to a library of benzisoxazolines (Scheme 11).^43^ Closely related aryne (3+2)‐cycloadditions with in situ generated nitrones have also been demonstrated for the three‐component coupling leading to benzisoxazolines.^44^ The reaction of nitrones with D–A cyclopropanes proceeded in an analogous fashion, but resulted in the formation of six‐membered rings via a (3+3)‐cycloaddition. Using Yb(OTf)_3_ as the Lewis acid, the Kerr group uncovered a mild method for the (3+3)‐cycloaddition leading to the diastereoselective synthesis of tetrahydro‐1,2‐oxazines **41**.^45^ A three‐component (3+3) cycloaddition via in situ generation of nitrones from the corresponding aldehydes and *N*‐aryl hydroxylamine proved also to be successful.^46^

**Scheme 12:**
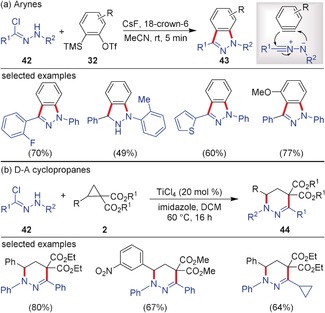
a) (3+2)‐Cycloaddition of arynes and b) (3+3)‐cycloaddition of D–A cyclopropanes with nitrile imines.

A similar reactivity profile ((3+2) vs. (3+3)) was observed with in situ generated nitrile imines from hydrazonyl chlorides **42**. The (3+2)‐cycloaddition of nitrile imines with arynes leading to the synthesis of 1*H*‐indazoles **43** was reported by Moses and co‐workers.^47^ This reaction was completed in just 5 minutes resulting in a convergent and rapid access to 1*H*‐indazoles in moderate to excellent yields (Scheme 12).^47^ In this case CsF was used for the generation of benzyne as well as for the generation of the nitrile imine from the precursor **42**. Parallel to the reactivity with arynes, the reaction of in situ generated nitrile imines with D–A cyclopropanes afforded tetrahydropyridazines **44** in high yields. Werz and co‐workers reported that D–A cyclopropanes activated by TiCl_4_ and nitrile imines generated from **42** using imidazole undergo smooth (3+3)‐annulation resulting in the formation of the diverse pyridazine derivatives, which are difficult to synthesize by other methods.^48^ A series of D–A cyclopropanes and multi‐substituted hydrazonyl chlorides are well tolerated in this annulation reaction.

**Scheme 13:**
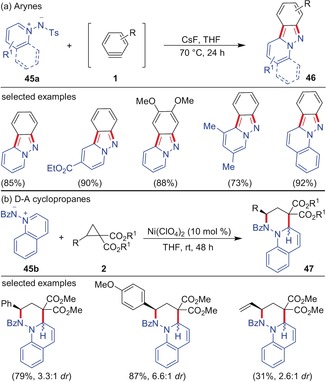
a) (3+2)‐Cycloaddition of arynes and b) (3+3)‐cycloaddition of D–A cyclopropanes with azomethine imines. Bz=benzoyl, Ts=*p*‐toluenesulfonyl (Tosyl).

Aromatic azomethine imines also showed similar reactivity towards arynes and D–A cyclopropanes. Wu, Shi and co‐workers observed that a series of *N*‐tosyl pyridinium imides **45 a** undergo smooth (3+2)‐cycloaddition with arynes resulting in the formation of pyridoindazoles **46** under mild and operationally simple conditions (Scheme 13).^49^ The initially formed cycloadduct eliminates a sulfinate anion to form the thermodynamically more stable, fully conjugated product **46**. Regardless of the substitution pattern, the products were formed in high yields. Concerning the reactivity of D–A cyclopropanes, Charette and co‐workers reported an analogous (3+3)‐annulation of a variety of quinoline‐derived azomethine imines **45 b** with cyclopropanes catalyzed by Ni(ClO_4_)_2_ affording the tricyclic dihydroquinolines **47** in good yield and moderate diastereoselectivity.^50^ A non‐concerted mechanism was proposed for this transformation and the reaction worked well with enantiomerically pure cyclopropanes leading to the inversion of stereochemistry at the cyclopropane carbon. Later, the enantioselective version of this (3+3)‐annulation reaction using a chiral ligand was demonstrated by Tang and co‐workers.^51^

In addition, the 1,3‐dipolar cycloaddition of arynes with other 1,3‐dipoles such as diazo compounds,^52^ nitrile oxides,^53^ and sydnones^54^ have been well demonstrated. However, there have been no reports on related (3+3)‐annulation processes of these dipoles with D–A cyclopropanes.

### 3.3 Reaction with Aldehydes and Imines

**Scheme 14:**
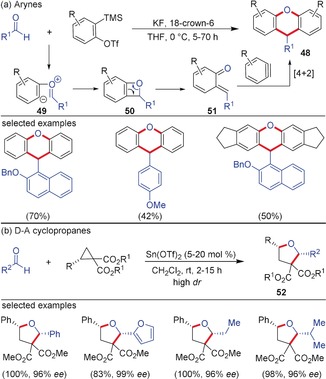
a) Cascade reaction of arynes and b) (3+2)‐annulation of D–A cyclopropanes with aldehydes.

Owing to the high electrophilicity of arynes, even electrophiles such as aldehydes and imines are able to add to these species. In 2004 Yoshida, Kunai and co‐workers reported the reaction of arynes with electron‐rich/neutral aldehydes (based on a 2:1 coupling) resulting in the formation of 9‐arylxanthene derivatives **48** in a one‐step operation. This reaction proceeds in a unique cascade process involving a [2+2]/retro‐[2+2]/[4+2] sequence resulting in the product formation.^55^ The nucleophilic attack of aldehydes on arynes generates the zwitterion **49**, which undergoes cyclization to generate the adduct **50** (formal [2+2]‐cycloaddition of aldehydes to arynes). The [2+2]‐adduct is unstable and undergoes electrocyclic ring‐opening in a retro‐[2+2] fashion to form *o*‐quinone methide intermediate **51** (Scheme 14). This intermediate is able to add to an excess of aryne resulting in the formation of the product. A closely related annulation using cyclopropenones instead of aldehydes afforded the spirocyclic xanthene–cyclopropene scaffolds.^56^ In contrast, the reaction of D–A cyclopropanes with aldehydes resulted in the formation of stable (3+2)‐annulated products. These annulations had been initially limited to alkoxy‐substituted cyclopropanes as donors.^57^ In 2005, the Johnson group uncovered the Sn‐catalyzed enantiospecific (3+2)‐annulation of chiral aryl‐ and alkyl‐substituted D–A cyclopropanes with aldehydes resulting in the formation of 2,5‐*cis*‐disubstituted tetrahydrofurans **52** in high diastereoselectivity.^58^ Mechanistically, this reaction proceeds via an analogous nucleophilic addition of aldehydes to activated cyclopropanes in a S_N_2 fashion, which explains the stereochemical outcome of the reaction.^59^ Related (3+2)‐annulations of amino‐substituted D–A cyclopropanes with aldehydes leading to the diastereoselective synthesis of aminotetrahydrofurans were reported by Waser and co‐workers.^60^ Moreover, similar to arynes, cyclopropenones were also used as carbonyl coupling partners in the (3+2)‐annulation furnishing spirotetrahydrofurans in good yields.^61^ Although two very different types of products are formed in the reaction with carbonyls there are striking similarities in the reaction mechanism. The reason why different pathways are followed can be traced back to the sharp difference in strain after formation of the four‐ and the five‐membered rings, respectively. The benzoxetane formed in the reaction of benzynes with aldehydes paves the way for a facile ring‐opening. The emerging intermediate shows heterodiene‐like reactivity with a second aryne (cf. Section 3.1).

**Scheme 15:**
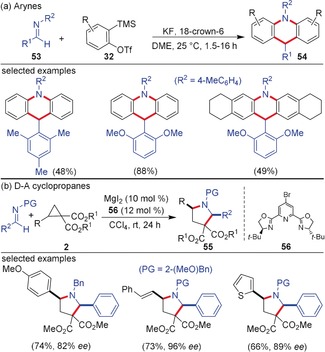
a) Cascade reaction of arynes and b) (3+2)‐annulation of D–A cyclopropanes with imines. PG=protecting group.

Imines showed a closely related reactivity profile with arynes and D–A cyclopropanes. Recently, Yoshida and co‐workers reported the 2:1‐coupling of arynes with aldimines **53** for the synthesis of functionalized acridanes **54** (Scheme 15).^62^ This reaction is an aryne‐imine‐aryne coupling following the [2+2]/retro‐[2+2]/[4+2] sequence (cf. Scheme 14); the key intermediate in this case is the aza‐ *o*‐quinone methide generated by the initial [2+2]‐cycloaddition followed by a retro‐[2+2] pathway. D–A cyclopropanes also resemble arynes in their reactivity towards imines. The Lewis acid‐catalyzed interception of aldimines with activated cyclopropanes in a (3+2)‐annulation for the synthesis of tetrahydropyrroles was pioneered by Carreira and co‐workers.^63^ Later, a Yb(OTf)_3_‐catalyzed three‐component reaction of aldehydes, amines, and D–A cyclopropanes for the diastereoselective synthesis of pyrrolidines **55** was demonstrated by the Kerr group.^64^ In 2010, Johnson and co‐workers reported the dynamic kinetic asymmetric synthesis of 2,5‐*cis*‐disubstituted pyrrolidines from racemic D–A cyclopropanes and aldimines. Catalyzed by MgI_2_ and using **56** as the chiral ligand, a series of D–A cyclopropanes and aldimines are well tolerated under the reaction conditions (Scheme 15).^65^

### 3.4 Reaction with Nitroso Compounds

**Scheme 16:**
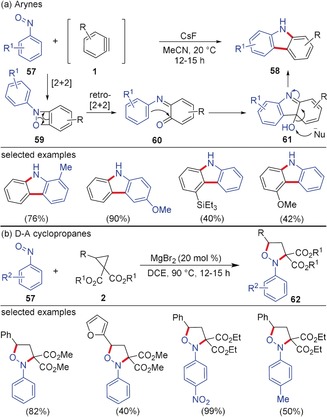
a) Cascade reaction of arynes and b) (3+2)‐annulation of D–A cyclopropanes with nitrosoarenes.

Even though the products of reactions of nitroso compounds with arynes and D–A cyclopropanes, respectively, look quite different at the first glance, there is a striking resemblance in their reactivity. Studer and co‐workers reported the reaction of arynes with nitrosobenzenes **57** resulting in the formation of carbazole derivatives **58** (Scheme 16).^66^ Depending on the fluoride source and the solvent employed, either N−H carbazole (CsF in MeCN) or *N*‐aryl carbazole (Bu_4_N/Ph_3_SiF_2_ (TBAT) in dimethoxyethane (DME)) can be synthesized. The initial [2+2]‐annulation of arynes with **57** generates the adduct **59**, which undergoes a [2+2]‐cycloreversion to form aza‐*o*‐quinone methide **60**; a subsequent intramolecular electrophilic aromatic substitution forms the intermediate **61**. According to the authors, the C−O bond cleavage by a nucleophile (most likely the fluoride) afforded the N−H carbazole **58**. Interestingly, D–A cyclopropanes underwent smooth (3+2)‐annulation with nitrosoarenes resulting in the formation of structurally diverse isoxazolidines **62** in high yields and regioselectivity.^67^ The reaction was catalyzed by MgBr_2_ and the reaction proceeds with excellent stereospecificity with retention of stereochemistry by using enantiomerically highly enriched D–A cyclopropanes. The MgBr_2_ was crucial for this reaction as it forms the activated MgBr_2_‐complexed cyclopropane, bromide is assumed to open the cyclopropane in an S_N_2‐like fashion.

### 3.5 Reaction with Alkenes

**Scheme 17:**
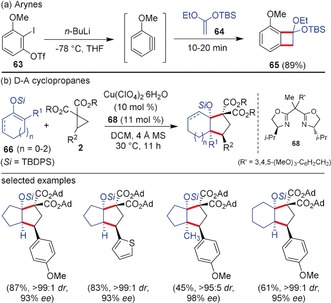
a) (2+2)‐Cycloaddition of arynes and b) (3+2)‐annulation of D–A cyclopropanes with electron‐rich alkenes. Ad=adamantyl, MS=molecular sieves, TBDPS=*tert*‐butyldiphenylsilyl.

Both arynes and D–A cyclopropanes react with alkenes to form annulated products. Because of the high electrophilicity of arynes and D–A cyclopropanes, usually these reactions proceed most efficiently with electron‐rich olefins. In contrast to the reactions of arynes with aldehydes/imines where the [2+2]‐adduct is not isolable, the [2+2]‐adducts of arynes with olefins are stable and isolable. Suzuki and co‐workers reported the reaction of 3‐methoxy benzyne generated from the precursor **63** with silyl enolethers **64** resulting in the facile, divergent, and regioselective synthesis of oxygenated benzocyclobutane derivatives **65**, which are promising candidates for accessing polycyclic aromatic compounds (Scheme 17).^68^ Moreover, the same group reported the consecutive three‐fold (2+2)‐cycloaddition of ketene silylacetals with arynes for the synthesis of polyoxygenated tricyclobutabenzenes.^69^ Similarly, polarized, electron‐rich olefins such as silyl enolethers and enamines are known to add to D–A cyclopropanes in a (3+2)‐pathway resulting in the formation of five‐membered carbocycles. The Cu‐catalyzed (3+2)‐annulation of D–A cyclopropanes with silyl enolethers for the diastereoselective synthesis of cyclopentanes has been reported by Tang and co‐workers.^24^ Related (3+2)‐annulations using chiral aminocyclopropanes resulting in the enantiospecific cyclopentane syntheses were realized by Waser and co‐workers.^70^ In 2013, Tang et al. disclosed an enantioselective (3+2)‐annulation of cyclic silyl enolethers **66** with D–A cyclopropanes for the synthesis of functionalized cyclopentanes **67** in high yields (Scheme 17).^71^ The use of a Cu^II^ catalyst along with ligand **68** was crucial for this transformation and several five‐ to seven‐membered cyclic ketone‐derived silyl enolethers were well tolerated.

## 4 Divergent Reactivity of Arynes and D–A Cyclopropanes

**Scheme 18:**
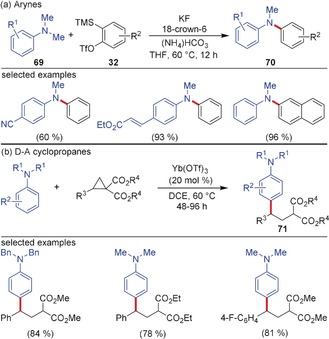
Divergent reactivity of aromatic tertiary amines with a) arynes and b) D–A cyclopropanes.

Although there are many parallels in the reactivity of arynes and D–A cyclopropanes and their respective transformations, a few cases exist where these intermediates behave very differently. Primary and secondary amines are known to insert into arynes and D–A cyclopropanes, respectively, resulting in the formation of 1,2‐ and 1,3‐bisfunctionalized products. However, the reaction using tertiary amines showed a surprisingly different reactivity profile. In 2013, Biju and co‐workers developed a monoarylation of tertiary amines using arynes as the aryl source for the synthesis of diaryl amines. A series of tertiary amines **69** are smoothly converted into the functionalized diaryl amines **70**, and the reaction works well with dyes and D–A systems bearing the NMe_2_ group (Scheme 18).^72^ The reaction proceeds via the addition of **69** to the aryne generated from **32** followed by the protonation of the emerging aryl anion and a subsequent demethylation. Interestingly, the reactivity was completely different with D–A cyclopropanes. The Lewis acid‐catalyzed reaction of D–A cyclopropanes with aromatic tertiary amines afforded the diarylated product **71** in high yields via the Friedel–Crafts alkylation of the tertiary amines.^73^ The use of Yb(OTf)_3_ was crucial for the reactivity and a wide range of functional groups were well tolerated under the reaction conditions.

**Scheme 19:**
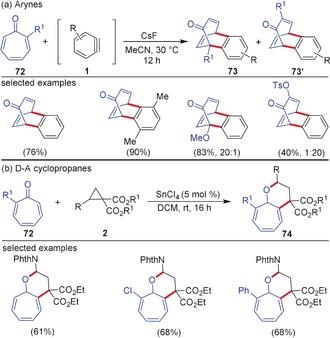
Divergent reactivity of tropones with a) arynes and b) D–A cyclopropanes. Phth=phthaloyl.

Moreover, tropones **72** also showed different reactivity towards arynes and D–A cyclopropanes. The conjugated seven‐membered rings offer multiple reactive sites in cycloaddition reactions starting from 2π to 8π components. With arynes, tropones react as 4π component, thus leading to an efficient Diels–Alder reaction for the synthesis of benzobicyclo[3.2.2]nonatrienone derivatives **73** in good yields (Scheme 19).^74^ In the case of reactions using substituted tropones, the electron‐rich diene moiety of tropone acts as the 4π component, and the Diels–Alder reaction is regioselective. Interestingly, tropones react in an (8+3)‐fashion with D–A aminocyclopropanes leading to the synthesis of tetrahydrocyclohepta[*b*]pyrans **74** in high regioselectivity and diastereoselectivity.^75^ Detailed mechanistic studies indicated that the (8+3)‐cycloaddition proceeds in a stepwise manner via an aromatic zwitterionic intermediate.

## 5 Conclusion and Outlook

The last two decades have witnessed a resurgence of interest in the chemistry of arynes and D–A cyclopropanes. On one hand, arynes are highly electrophilic intermediates commonly utilized for the synthesis of diverse 1,2‐disubstituted benzene derivatives. On the other hand, D–A cyclopropanes as masked 1,3‐dipoles have emerged as versatile three‐carbon building blocks for organic synthesis. Although arynes are short‐lived intermediates, whereas D–A cyclopropanes are stable compounds there are numerous similarities in reactivity. In brief, these similarities can be traced back to the fact that (i) both systems show a high strain energy providing a reasonable driving force and that (ii) one finds either an easily polarizable or an already polarized system. Arynes usually result in a 1,2‐disubstitution, whereas D–A cyclopropanes give rise to a 1,3‐bisfunctionalization. Furthermore, numerous annulation reactions show astonishing similarities; the three‐membered rings often lead to analogous cyclic systems being one carbon atom larger than in the case of arynes. Because D–A cyclopropanes lead to sp^3^‐hybridized carbon atoms in contrast to the sp^2^‐hybridized carbons emerging from arynes the degree of unsaturation or conjugation in the respective products is commonly different.

An attentive reader might also have noticed that several reactions that are known in the literature either for arynes or D–A cyclopropanes have not found their parallels in their respective counterparts yet. Of course, it is difficult to interpret such observations. Either the respective experiments have never been conducted or they have been conducted, but have not provided any successful results. Even the latter possibility might be traced back to the fact that the conditions and/or catalytic systems required to access the anticipated structures have not been found. Thus, this Minireview can be considered a stimulus for synthetic organic chemists to foster new ideas in aryne and D–A cyclopropane chemistry because their reactivities might be regarded as two sides of the same coin. Novel methodologies which have already been established for one of the two classes might be realized for the other as well. Interestingly, there are several challenges yet to be uncovered. For instance, there are quite a few 1,2‐bisfunctionalization reactions known with arynes for which the corresponding 1,3‐bisfunctionalization using D–A cyclopropanes have not been well studied. The insertion of arynes into C−C single bonds is widely known, whereas the related insertion across D–A cyclopropanes has not been realized. Moreover, the Diels–Alder reaction of arynes with dienes such as pentafulvenes and 1,2‐benzoquinones are known, but related (4+3)‐annulation processes using D–A cyclopropanes have not been studied. It is anticipated that the mild and straightforward routes for building molecular complexity using arynes and D–A cyclopropanes will continue to inspire a broad range of synthetic chemists to explore new applications and demonstrate unforeseen possibilities using these intermediates.

## Conflict of interest

The authors declare no conflict of interest.

## Biographical Information

***Daniel B. Werz** (left) received his diploma (2000) and Ph.D. (2003*, *R. Gleiter) from the University of Heidelberg supported by a scholarship of the Studienstiftung des deutschen Volkes. After a postdoctoral stay at the ETH Zurich (P. H. Seeberger) he began his independent research at the University of Göttingen in 2006 (mentor of the habilitation: L. F. Tietze). In 2013 he was appointed as associate professor of organic chemistry at TU Braunschweig where he was promoted to full professor in 2018. His research interests include donor–acceptor cyclopropanes, catalysis, carbohydrates, and organic dyes. **Akkattu T. Biju** (right) received his Ph.D. under the guidance of Dr. Vijay Nair at the CSIR‐NIIST (Formerly RRL), Trivandrum, India. Subsequently, he has been a post‐doctoral fellow with Prof. Tien‐Yau Luh at the National Taiwan University, Taipei and an Alexander von Humboldt fellow with Prof. Frank Glorius at the WWU Münster, Germany. In June 2011, he began his independent research career at the CSIR‐National Chemical Laboratory, Pune. From June 2017 onwards, he has been an Associate Professor at the Department of Organic Chemistry, Indian Institute of Science, Bangalore. His research focuses on the development of transition‐metal‐free carbon–carbon and carbon–heteroatom bond‐forming reactions and their application in organic synthesis*.